# The 2017 Dutch Physical Activity Guidelines

**DOI:** 10.1186/s12966-018-0661-9

**Published:** 2018-06-25

**Authors:** Rianne M. Weggemans, Frank J. G. Backx, Lars Borghouts, Mai Chinapaw, Maria T. E. Hopman, Annemarie Koster, Stef Kremers, Luc J. C. van Loon, Anne May, Arend Mosterd, Hidde P. van der Ploeg, Tim Takken, Marjolein Visser, G. C. (Wanda) Wendel-Vos, Eco J. C. de Geus, Committee Dutch Physical Activity Guidelines 2017

**Affiliations:** 1 Health Council of the Netherlands, P.O. Box 16052, 2500 BB The Hague, The Netherlands; 2 University Medical Center, Utrecht, the Netherlands; 3 Fontys University of Applied Sciences, Eindhoven, The Netherlands; 4 VU University Medical Center, Amsterdam, The Netherlands; 5 Radboud University Medical Center, Nijmegen, The Netherlands; 6 Maastricht University, Maastricht, The Netherlands; 7 Maastricht University Medical Center+, Maastricht, The Netherlands; 8 Meander Medical Center Amersfoort, Amersfoort, The Netherlands; 9 Vrije Universiteit Amsterdam and VU University Medical Center, Amsterdam, The Netherlands; 10 National Institute for Public Health and the Environment, Bilthoven, The Netherlands

**Keywords:** Guidelines, Physical activity, Chronic diseases, Fitness, Prospective cohort study, Randomized-controlled trial, Systematic review

## Abstract

**Background:**

The objective of this study was to derive evidence-based physical activity guidelines for the general Dutch population.

**Methods:**

Two systematic reviews were conducted of English language meta-analyses in PubMed summarizing separately randomized controlled trials and prospective cohort studies on the relation between physical activity and sedentary behaviour on the one hand and the risk of all-cause mortality and incidence of 15 major chronic diseases and conditions on the other hand. Other outcome measures were risk factors for cardiovascular disease and type 2 diabetes, physical functioning, and fitness. On the basis of these reviews, an expert committee derived physical activity guidelines. In deriving the guidelines, the committee first selected only experimental and observational prospective findings with a strong level of evidence and then integrated both lines of evidence.

**Results:**

The evidence found for beneficial effects on a large number of the outcome measures was sufficiently strong to draw up guidelines to increase physical activity and reduce sedentary behaviour, respectively. At the same time, the current evidence did not provide a sufficient basis for quantifying how much physical activity is minimally needed to achieve beneficial health effects, or at what amount sedentary behaviour becomes detrimental. A general tenet was that at every level of current activity, further increases in physical activity provide additional health benefits, with relatively larger effects among those who are currently not active or active only at light intensity. Three specific guidelines on (1) moderate- and vigorous-intensity physical activity, (2) bone- and muscle-strengthening activities, and (3) sedentary behaviour were formulated separately for adults and children.

**Conclusions:**

There is an unabated need for evidence-based physical activity guidelines that can guide public health policies. Research in which physical activity is measured both objectively (quantity) and subjectively (type and quality) is needed to provide better estimates of the type and actual amount of physical activity required for health.

**Electronic supplementary material:**

The online version of this article (10.1186/s12966-018-0661-9) contains supplementary material, which is available to authorized users.

## Background

Physical activity and sedentary behaviour guidelines provide guidance on how the general population can improve its health through physical activity. The recommendations in these guidelines are based on a meticulous review of the scientific knowledge available in the international scientific literature. Most existing guidelines are based on evidence from both prospective and cross-sectional studies [1–6]. The aim of the Committee for the Dutch Physical Activity Guidelines 2017 was to derive physical activity guidelines based on the integration of evidence from various kinds of prospective studies, i.e. studies in which physical activity and/or sedentary behaviour was assessed before the outcome was measured. Information from prospective cohort studies (prospective cohort studies, nested case-control studies, and case-cohort studies) was integrated with that of RCTs. These types of studies provide stronger evidence for causality than cross-sectional studies [7]. This paper describes the methods of derivation, the main results of this effort and a full description of the guidelines [8].

## Methods

A multidisciplinary committee of 14 scientists was appointed, who filled out a declaration of interest published on the website of the Health Council (www.gezondheidsraad.nl). First, a methodology document was prepared, that describes the methods used for the evaluation of the evidence [9]. Based on this methodology the committee supplemented the evidence from existing guidelines with more recently published evidence on the effects of physical activity, endurance and/or strength training and sedentary behaviour on premature mortality risk and the risk of 15 major physical-activity related chronic diseases and other conditions (Table 1). For RCTs the committee also selected cardiometabolic risk factors and fitness indicators as outcomes.

**Table 1:** Exposure and outcome measures

Main exposures:	
Physical activity, endurance training, strength training, balance training, sedentary behaviour and TV-watching time	
Outcome measures:	
In prospective cohort studies and RCTs^a^	In RCTs
Coronary heart disease	Systolic blood pressure
Stroke	LDL cholesterol
Heart failure	Body weight (adults) and body mass index (children)
Type 2 diabetes mellitus	Insulin sensitivity
Chronic obstructive pulmonary diseases	Blood glucose
Breast cancer	Fat mass
Colorectal cancer	Abdominal fat
Lung cancer	Waist circumference
Disability	Fat-free mass
Fractures	Bone density
Osteoarthritis	Cardiorespiratory fitness
Musculoskeletal injuries	Functional performance
Dementia and cognitive decline	Muscle strength
Depression and depressive	
symptoms	
ADHD symptoms	

^a^Meta-analyses of RCTs were encountered only for type 2 diabetes, fractures, musculoskeletal injuries, cognitive decline and depressive symptoms

For each outcome measure, the committee used the systematic reviews of the evidence in the most recent guidelines, i.e. the Australian reports [10–12] as a starting point. If the Australian reports did not include any conclusions regarding particular outcome measures, the older report describing the evidence for the US guidelines was additionally used [1]. Next the committee searched the literature for studies on the effects of physical activity and sedentary behaviour published in peer-reviewed journals between 2012 up to October 2016 in PubMed, i.e. after publication of the Australian reports. The literature search of the committee for the newer scientific evidence was restricted to pooled analyses, meta-analyses and systematic reviews of RCTs or prospective cohort studies. Only when no pooled/meta-analyses or systematic reviews were available, individual studies were used (for example, RCT regarding the effect of physical activity on the risk of diabetes). In addition, the single cohort study that included objective measurement of physical activity has been described separately, because this measurement is more reliable than the use of self report [13, 14].

Systematic reviews and meta-analyses were selected that summarized studies in the general population, with samples spanning the entire lifespan, i.e. from childhood to old age. However, we excluded studies that exclusively used clinical samples or studies in pregnant or lactating women. An exception was made for RCTs in prediabetic, overweight or hypertensive individuals as the prevalence of these conditions in the general population is high.

The committee evaluated the evidence on the health effects of physical activity and sedentary behaviour taking into account the availability of the research, the strength of the associations and the presence of heterogeneity in meta-analysis. We used the decision tree in Additional file 1 when drawing conclusions about the strength of the evidence. On the basis of the experiences of the Health Council with devising a methodology for the 2015 Dutch Dietary Guidelines [15], the committee derived the criteria for the required number of studies and participants for each type of conclusion. The conclusion that the level of evidence is strong or that an effect or association is unlikely implies that there are at least five studies involving 150 participants (RCTs) or 500 cases (cohort studies) with consistent findings; the conclusion that there is a weak level of evidence implies three or four studies and at least 90 participants (RCTs) or 300 cases (cohort studies); one or two studies means that the conclusion is that there is too little research. The required number of participants in individual RCTs naturally depends on the variation in outcome measure and the expected effect size. The experience of the committee is that, albeit arbitrary, these cut-off values are helpful in practice.

Strong evidence from cohort studies and RCTs was then integrated, as next step in the derivation of the guidelines. If the results of cohort studies on chronic diseases and at least one individual RCT with disease as end point were consistent, the committee regarded the support from the evidence as convincing. The committee rated the support from the evidence also convincing if the results of the cohort studies and RCTs with a cardiometabolic risk factor were consistent. Finally, a significant effect on a risk factor or indicator of fitness was also rated convincing. If only results of cohort studies were available, the committee judged the association plausible.

In the case of convincing support from both cohort studies and RCTs, the guideline was further quantified by a desirable amount of physical activity. We based these amounts of physical activity or sedentary behaviour on the amounts observed to yield health effects in cohort studies to ensure the attainability of the guidelines in practice. Where possible, quantification was supplemented with data from RCTs regarding the effective levels of intensity, frequency and duration of physical activity. Those conclusions with plausible support only do not provide a sufficient basis to derive quantitative guidelines.

## Results

Most studies relate to the effects in children aged four and older and in adults, whereas data on children up to the age of four years is scarce. The committee provides guidelines for physical activity, muscle- and bone-strengthening physical activity, and for sedentary behaviour separately for children/adolescents from 4 to 18 and for adults. No further subdivision was made between the younger and older adult age span, as no meaningful cut-off age could be found to make such a distinction. The cohort studies in adults that were used for the guidelines often included older persons. For these older persons the evidence in adults can therefore be used with no further modifications. However, a number of additional outcomes were included for older adults: the risk of fractures, disability, and cognitive decline and dementia. The guidelines take this into account, by providing an additional recommendation specifically aimed at older adults.

As indicated in the methods, we used systematic reviews for derivation of the guidelines. In the text below, we use ‘cohort studies’ and ‘RCTs’ as a short form for meta-analyses and systematic reviews of cohort studies and RCTs, respectively.

In cohort studies, moderate and high-intensity physical activity is generally compared to no and light-intensity physical activity. In some studies there is no distinction between different intensities, but there is a high amount of physical activity compared to a low amount. In RCTs, usually either endurance or strength training (resistance-type exercise) programmes are studied or a combination of the two.

Physical (in)activity and sedentary behaviour are different kinds of concepts. Physical inactivity refers to an insufficient physical activity level to meet physical activity guidelines. Sedentary behaviour is any waking behaviour characterized by an energy expenditure ≤ 1,5 metabolic equivalents, while in a sitting, reclining or lying posture [16]. In cohort studies on sedentary behaviour, a high amount of time spent in sedentary behaviour is often compared with a low amount with adjustment for physical activity. Therefore, in studies on physical activity and on sedentary behaviour different comparisons are made.

### Adults: health effects of physical activity

#### Physical activity and risk of cardiovascular disease

There is convincing evidence that physical activity reduces the risk of cardiovascular disease (Table 2) [17]. Cohort research reveals an association between a high level of physical activity and a reduced risk of cardiovascular disease [18–20]. This is supported by RCTs that demonstrate that endurance training and strength training reduce blood pressure [21–23]. In addition, endurance training also reduces fat mass and abdominal circumference [22, 24–26].

**Table 2:** Summary of strong evidence for health effects of physical activity and sedentary behaviour

Health effects of physical activity		
Population group	Cohort research Physical activity is associated with a lower risk of:	RCTs Physical activity has a beneficial effect on:
Adults		
Convincing	Depressive symptoms	Depressive symptoms
	Cardiovascular disease	Blood pressure Fat mass Abdominal circumference
	Diabetes	Weight Insulin sensitivity Diabetes (1 study)
* Plausible*	*Breast cancer*	
	*Colorectal cancer*	
	*Premature mortality*	
Older adults		
Convincing	Fractures, especially hip fractures	Fractures
		Muscle strength
		Fat-free mass
		Walking speed
* Plausible*	*Dementia, cognitive decline and Alzheimer’s disease*	
	*Disability*	
Children		
Convincing	Depressive symptoms	Depressive symptoms
		Cardiorespiratory fitness
		Muscle strength
		Insulin sensitivity
		Weight and fat mass in children with overweight or obesity
		Bone quality
Health effects of sedentary behaviour		
Population group	Cohort research Sedentary behaviour is associated with an increased risk of:	
Adults		
* Plausible*	*Death from cardiovascular disease*	
	*Premature mortality*	

The cohort research on cardiovascular disease provides some indication of the required amount and intensity of the physical activity. The key finding is: the more physical activity, the greater the beneficial effects. In relative terms, the greatest benefit can be achieved when a physically inactive person becomes active, i.e. engages in sufficient physical activity of at least moderate intensity: research shows that 75 minutes a week of moderately intensive physical activity reduces the risk of heart attack and heart failure; at 150 minutes a week, the risk decreases further, and at 300 minutes or more the effect is even more beneficial [18, 19]. The studies on stroke also show a beneficial effect of physical activity of moderate and vigorous intensity [20].

RCTs on cardiovascular outcomes confirm the importance of endurance training of moderate and vigorous intensity and of strength training [21–23]. The typical frequency of the strength training was three to five times a week, using the muscles of the hands or legs four times for two minutes. It was not possible to draw any conclusion regarding the amount of physical activity required based on the RCTs, because the variation between the studies in frequency and duration of the endurance training and the intensity of the strength training was large.

#### Physical activity and risk of diabetes

Convincing evidence was found that more physical activity reduces the risk of diabetes [17]. Cohort research shows an association [27, 28] that is supported by findings from RCTs. For example, endurance training and strength training have been shown to improve whole body insulin sensitivity [29, 30]. Endurance training also reduces body weight in adults with normal weight, excess weight and obesity [1, 22, 24, 25, 31, 32]. Finally, one specifically designed RCT has shown that physical activity reduces the risk of diabetes [33]. How much physical activity is required to reduce the risk of diabetes is not known on the basis of the cohort studies because the amount of physical activity was not quantified sufficiently [27, 28]. Neither do the RCTs give a good indication. RCTs show that moderate to vigorous-intensity endurance training has a beneficial effect, but the variation in training frequency (three to six times a week) and duration (24 to 90 minutes per session) was too large to determine how much physical activity is needed [22, 24, 25, 29–32]. RCTs that examined strength training found favourable effects for two to three training sessions a week at moderate to vigorous intensity. Again, the available data is too limited to determine the minimal or optimal dose for health benefits [29]. In the only RCT examining the effect of physical activity on diabetes, the training programmes used (30 to 60 minutes of light activity per day to five to ten minutes of vigorous-intensity activity per day) differed too widely to quantify the amount of physical activity required [33].

#### Physical activity and depressive symptoms

The effect of physical activity on the risk of depressive symptoms is also convincing [17]: cohort research shows an association between physical activity and lower risk of depressive symptoms [1, 34]. This is supported by RCTs showing that endurance training at moderate to vigorous intensity and strength training reduce the risk of depressive symptoms [35]. As with diabetes, based on the current evidence, it is not possible to quantify how much physical activity is needed to achieve the beneficial effect.

#### Premature death, breast cancer and colorectal cancer

Cohort research has found an association between physical activity and a reduced risk of premature death, breast cancer and colorectal cancer [17, 36–42]. This makes it plausible that there is indeed an association. For premature mortality and breast cancer, there are indications that the greatest relative benefit is achieved when inactive persons (during leisure time) become active. Higher levels of physical activity are associated with further health gains [36–41].

#### Older adults: fractures, disability and dementia and cognitive decline

There is convincing evidence that physical activity reduces the risk of fractures in older persons [17]. Cohort studies show that higher levels of physical activity are associated with a lower risk of fractures in general and hip fractures in particular [43, 44], while RCTs demonstrate that the combination of endurance and strength training and/or balance exercises reduce the risk of fractures [45].

The cohort research on fractures does not provide an indication of the amount of physical activity required, as this was insufficiently quantified in the studies [43, 44]. In the RCTs looking at the combination of endurance training and strength training, the endurance training was of moderate to vigorous intensity. However, the frequency (one to seven times a week), duration (20 to 60 minutes per session), type of strength training and intensity (light to vigorous) varied too much to make a conclusive statement about the amount required [45].

There is convincing evidence that strength training improves walking speed and muscle strength [17]. There are RCTs that show that strength training increases walking speed in older persons [46, 47]. RCTs also show that strength training increases muscle strength and fat-free mass in older persons [48, 49].

In the RCTs, the beneficial effects on walking speed were found for strength training two to three times a week with 45 to 60 minute sessions [46, 47]. Strength training two to three times a week at light to moderate intensity increases the fat-free mass [48] and the beneficial effect on muscle strength increases with the intensity of strength training [49, 50]. These studies do not provide data about the number of exercises and the number of times they are performed for each training, and therefore do not provide a basis for a statement about the quantification of physical activity needed to achieve the beneficial effect.

It is plausible that physical activity is associated with a lower risk of disability [17]. Cohort research finds such a link with a moderate to high level of physical activity [51].

As shown by cohort studies [52, 53], it is also plausible that higher levels of physical activity in older persons are associated with a lower risk of cognitive decline, dementia and Alzheimer's disease.

### Adults: guidelines physical activity

#### Guideline: physical activity is good for you – the more, the better

A general guideline was derived, in view of the fact that the systematic review of the literature reaffirms the numerous beneficial effects of regular physical activity [Table 2). The key finding is: the more physical activity, the greater the health benefit. Where the recommendations are not attainable, any physical activity is better than none, and this applies to everybody.

#### Guideline: Engage in physical activity of moderate intensity for at least 150 minutes every week, spread over several days. For example, walking and cycling. The longer you are physically active, and the more frequent and/or more intensive the activity, the more your health will benefit

The committee concludes that research in adults provides grounds for recommending at least 150 minutes of physical activity per week at moderate intensity, spread over several days. A more exact amount of physical activity required for health benefits could not be distilled from the evidence, because in many studies the amount of physical activity was insufficiently quantified. The choice for 150 minutes reflects a deliberate choice for continuity with existing guidelines: we had insufficient grounds to deviate from this widely used amount.

The recommendation to spread the physical activity over several days is predicated on the fact that most RCTs used physical activity programmes that repeat exercises on more than one occasion a week [17]. This gives us confidence that spreading activity over more than one day produces certain benefits, but it does not refute that other schemes could also work. In short, the committee sees no scientific basis for the recommendation to spread the 150 minutes over at least five days a week, nor for recommending continuous bouts of activity that span at least 10 minutes, as required by the current Dutch Norm for Healthy Physical Activity and several other guidelines [2–4, 17, 54–57].

We found convincing evidence that higher amounts of physical activity, in terms of the net product of duration, frequency and/or intensity provide added health benefits, although there appears to be a diminishing gain of extra physical activity at higher levels of one’s current physical activity.

#### Guideline: Engage in activities that strengthen your muscles and bones at least twice a week. Older people should combine these with balance exercises

As indicated before, the committee has chosen one single guideline for adults and older persons because much adult research includes older persons. The research carried out specifically in older persons confirms the required intensity (moderate to vigorous). Nonetheless, older persons experience additional health gains from specific physical activities targeting balance and strength.

The committee concludes that there is convincing evidence for the benefits of muscle and bone-strengthening exercises in general, and the addition of balance exercises for older persons. Because in most RCTs these exercises were carried out two to three times per week, the committee recommends a frequency of at least twice a week [17]. This corresponds to international physical activity guidelines for muscle-strengthening activities (involving large muscle groups) at least twice a week. Some guidelines also recommend bone-strengthening exercises. Exercises that focus on balance and flexibility are sometimes covered by these guidelines and sometimes under the guidelines for persons with an increased risk of falling [2–4, 56–59].

### Adults: health effects of sedentary behaviour

It is plausible that sedentary behaviour is associated with a higher risk of premature death and death from cardiovascular disease [60]. Cohort research shows this association for sedentary behaviour (more than eight hours a day compared to less than four hours a day). This association becomes weaker the more physical activity that people engage in, and is not present in those with a high level of physical activity (significantly more than the norm for physical activity) [61]. The scientific evidence for the health effects of sedentary behaviour is currently much weaker than that for physical activity, both in adults and older persons, and in children (Table 2).

### Adults: guideline sedentary behaviour

#### Guideline: avoid long periods sitting down

The research evaluated allows a qualitative recommendation but there is not yet enough data to make a quantitative recommendation. Recent guidelines from Flanders, France, Germany, Australia and Great Britain now advise adults to limit the time that they spend sedentarily. The Flemish guideline recommends interrupting sitting every 30 minutes [59], while the French guideline [62] recommends doing so every 90 to 120 minutes. The variations in international guidelines illustrate that research into the health effects of sedentary behaviour is still emerging [3, 4, 56, 58, 59, 62].

### Children and adolescents: health effects of physical activity

For children most of the (disease) outcomes used for the adults are not yet relevant. However, substantial literature was found for intermediate risk factors, fitness indicators and mental health problems.

#### Physical activity and depressive symptoms

There is convincing evidence that physical activity reduces the risk of depressive symptoms [17]. Cohort research finds an association between increased physical activity in children and a lower risk of depressive symptoms [1, 34, 63] and RCTs show that endurance training in children with an increased risk of these symptoms reduces the chance that these will actually occur [64]. The level of physical activity required for a beneficial effect is not clear. The amount of physical activity was not quantified in cohort studies [1, 34, 63]. The RCTs involved moderate to vigorous intensity endurance training two to three times a week, but the variation in duration (20 to 90 minutes per session) is too large to draw a definitive conclusion on the amount required [64].

#### BMI, fat mass and insulin sensitivity

It has been demonstrated convincingly that physical activity reduces body mass index (BMI) and fat mass in children with overweight and obesity, after natural growth is accounted for [17, 65, 66]. No effects have been found in children with a normal weight [67–69]. RCTs have found that endurance training at moderate to vigorous intensities has a beneficial effect on BMI and fat mass in children with overweight and obesity, although these effects are small. The variation in the frequency and duration of the sessions is too large to say how much training is required [11, 64–66].

We also found convincing evidence that strength training during which body weight is used as resistance increases bone quality [17]. However, the required amount of strength training cannot be deduced from these RCTs [70]. The frequency, number of repetitions and duration of physical activity in the studies varied too much, and there was insufficient information about its intensity.

Finally, a combination of endurance and strength training improves insulin sensitivity [17, 71]. The duration was 40 to 90 minutes, carried out two to four times a week. Because no information is provided about the intensity of training, these RCTs are not sufficient to quantify the amount of training required [71].

#### Cardiorespiratory fitness and muscle strength

We found also convincing evidence that endurance training improves cardiorespiratory fitness in children and that strength training increases muscle strength in children [17]. This is evident from RCTs [11, 72, 73]. For an effect on cardiorespiratory fitness, a combination of moderate and vigorous-intensity exercise is required [11]. Here too, the RCTs do not provide any evidence regarding the amount of physical activity required [11, 72, 73].

### Children: physical activity and sedentary behaviour guidelines

*Guideline: Engage in physical activity of moderate intensity for at least one hour every day. The longer you are physically active, and the more frequent and/or more intensive the activity, the more your health will benefit.*

The often cited beneficial effects of physical activity in children on intermediate risk factors and fitness indicators were fully reaffirmed by our review of the recent literature (Table 2). The research evaluated does not, however, provide enough footing to derive a meaningful quantification for this physical activity. By the same token, it also did not provide grounds for adapting the current norm [17] so we again chose for continuity with existing guidelines. The committee therefore advises children to engage in moderate to high-intensity physical activity for at least one hour every day. This echoes the majority of international guidelines for this age range (at least one hour a day at moderate to vigorous intensity) with Germany being the only country to advise at least 90 minutes a day [2–4, 56–59].

*Guideline: Engage in activities that strengthen your muscles and bones at least three times a week.*

The research evaluated shows the beneficial effects of muscle and bone-strengthening activities. The research allows no conclusions regarding the number of times per week that is required. Neither did the committee find any research into the effects of exercises focusing on flexibility and coordination on risk factors and fitness [17]. Internationally, most countries recommend at least three times a week (as part of the high-intensity physical activity) [2–4, 57–59]; only the Dutch Norm for Healthy Physical Activity [54, 55] mentions twice a week while the German guidelines recommend two to three times a week [56]. The committee has chosen to concur with the majority of the international guidelines for physical activity, at least three times per week, further increasing the coherence of the guidelines across countries.

*Guideline: Avoid long periods sitting down.*

The recommendations for sedentary behaviour are entirely based on the adult research described above, neither plausible nor convincing evidence was encountered but this mostly reflects a paucity of research. To err on the side of caution, the committee extends the recommendation for adults to children aged four years and over [60].

### Children aged zero to four years

The committee found no research that provides a basis for establishing a recommendation for this age group [17]. International physical activity guidelines for this age group are based on opinions of experts and experience in practice [3, 4, 56, 58]. The Dutch Norm for Healthy Physical Activity includes no separate recommendations for young children [54, 55].

The committee has chosen to make no specific recommendations for this age group. It fully recognizes the importance for young children to engage in varied forms of physical activity to acquire the motor skills that are needed to become physically active after the age of four and onwards [74].

### Adverse effects of physical activity

It is striking that only limited research has been done into the risk of injuries when following a programme aiming to increase the level of physical activity [17]. The committee found weak evidence that a small proportion of people engaging in physical activity may suffer a slight injury, while it is unlikely that increased levels of physical activity increase the risk of serious injuries [75]. However, there are strong indications that the risk of injury is greater for contact sports than non-contact sports [1, 10]. A large problem in interpreting these ‘adverse effects’ of sports is that they are not based on the correct comparison of the total injury risk of exercisers and non-exercisers (i.e. injuries inside but also outside the sports and exercise context). The comparison that comes closest to this are the studies comparing effects of physical activity on musculoskeletal health. In these studies the committee found convincing evidence for a protective effect of more physical activity on the incidence of fractures [17, 43–45].

### How do the new guidelines relate to the current international and Dutch norms?

The new guidelines are largely similar to international guidelines and the previous Dutch norms [2–4, 54–59, 76, 77]. However, some differences should be noted (Tables 3 and 4). Compared to the WHO Physical Activity Guidelines for instance, there is no distinction between younger and older adults [2]. The recommendations for moderate and vigorous-intensity activities are combined in the new guidelines for this group and the minimum amount of physical activity required is not expressed per day, but per week, spread over a number of days. For all age categories applies that higher amounts of physical activity, in terms of the net product of duration, frequency and/or intensity provide added health benefits. Finally, the guideline related to sedentary behaviour is new. There are additional differences with the previous Dutch norms [54, 55, 76, 77].

**Table 3:** The 2017 Dutch, WHO [2] and original Dutch Physical Activity Guidelines [54, 55, 76, 77] for children and adolescents

2017 Dutch Physical Activity Guidelines	WHO Global Recommendations on Physical Activity for Health [2]	Original Dutch norms [54, 55, 76, 77]
Physical activity is good for you – the more, the better.		
Engage in physical activity of moderate intensity for at least one hour every day. The longer you are physically active, and the more frequent and/or more intensive the activity, the more your health will benefit.	Should do at least 60 minutes of moderate to vigorous-intensity physical activity daily. Physical activity of amounts greater than 60 minutes daily will provide additional health benefits.	At least one hour per day moderate to high intensity physical activity OR at least three times per week at least 20 minutes high intensity physical activity
Do activities that strengthen your muscles and bones at least three times a week.	Should include activities that strengthen muscle and bone, at least three times per week.	Of which two times per week activities focusing on muscle strength, agility, coordination and bone strength
And: avoid spending long periods sitting down.	No recommendation	No recommendation
The guidelines do not include a recommendation on a specific duration of bouts, as there was insufficient evidence.	In order to be beneficial for cardiorespiratory health, all activity should be performed in bouts of at least 10 minutes duration.	All activity should be performed in bouts of at least 10 minutes duration.

**Table 4:** The 2017 Dutch, WHO [2] and original Dutch Physical Activity Guidelines [54, 55, 76, 77] for adults and older persons

2017 Dutch Physical Activity Guidelines	WHO Global Recommendations on Physical Activity for Health [2]	Original Dutch norms [54, 55, 76, 77]
*Adults*	*Adults 18-65 years*	*Adults 18-55 years*
Physical activity is good for you – the more, the better.		
Engage in physical activity of moderate intensity for at least 150 minutes every week, spread over several days. For example, walking and cycling. The longer you are physically active, and the more frequent and/or more intensive the activity, the more your health will benefit.	Should do at least 150 minutes of moderate-intensity physical activity throughout the week, or do at least 75 minutes of vigorous-intensity physical activity throughout the week, or an equivalent combination of moderate- and vigorous-intensity activity. For additional health benefits, adults should increase their moderate-intensity physical activity to 300 minutes per week, or equivalent.	At least 5 days per week at least 30 minutes per day moderate intensity physical activity OR at least 3 times per week at least 20 minutes per day high intensity physical activity
Do activities that strengthen your muscles and bones at least twice a week. Older people should combine these with balance exercises.	Muscle-strengthening activities should be done involving major muscle groups on 2 or more days a week.	No recommendation
And: avoid spending long periods sitting down.	No recommendation	No recommendation
*Older adults*	*Adults 65+*	*Adults 55+*
Similar to the recommendations for younger adults above.	The above recommendations for younger adults in combination with: Those with poor mobility should perform physical activity to enhance balance and prevent falls, 3 or more days per week.	The above recommendations for younger adults with lower cut-off values for moderate and high-intensity physical activity
The guidelines do not include a recommendation on a specific duration of bouts, as there was insufficient evidence.	In order to be beneficial for cardiorespiratory health, all activity should be performed in bouts of at least 10 minutes duration.	All activity should be performed in bouts of at least 10 minutes duration.

## Discussion

The 2017 Dutch Physical Activity Guidelines provide advice on how much physical activity the population should adopt to achieve health gains. Based on the existing scientific literature, the committee judged the underpinning of the physical activity guidelines ‘convincing’; whereas the underpinning of the sedentary behaviour guideline is ‘plausible’. Substantial overall public health gains could be obtained if these guidelines were more widely adopted. Analyses by the National Institute of Public Health based on self report show that only 44% of Dutch adults and older persons currently meet the new physical activity guidelines in that they perform at least 150 minutes per week of physical activity at moderate intensity, spread over a number of days, and engage in muscle and bone-strengthening activities at least two days a week. Not more than slightly over 40 percent of children engage in physical activity at moderate to vigorous intensity an hour every day and in muscle and bone-strengthening activities at least three days a week [78]. In addition, there are many people in the Netherlands who spend a lot of time sedentarily [79].

Despite the convincing substantiation of the general tenet in the physical activity guidelines that “more is better”, the data did not provide sufficient information to quantify the actual amount of physical activity that is minimally needed for an effect on any health outcome, or what the optimal amount would be across all outcome measures considered. Definitions and cut-off values for categories of physical activity and sedentary behaviour vary widely across the cohort studies summarized in meta-analyses and systematic reviews on which we relied for the guidelines. To illustrate this point, most studies based on self report only asked about leisure-time physical activity, not about overall physical activity. Hence, the committee considers the data insufficient to determine whether the associations that apply to leisure-time physical activity also apply to other forms of physical activity, such as household work, other forms of work or transport. Also in RCTs the amount of physical activity prescribed varied widely, with the only stable element being that multiple weekly exercise sessions were used in most interventions. However, duration, frequency and intensity varied substantially. Finally, screen time or time spent watching television have often been used as a proxy for sedentary behaviour, whereas the formal definition includes any waking activities performed in a sitting, reclining or lying posture, with low energy expenditure (≤1.5 MET), excluding sleep [16].

The varying operationalisations make it difficult to extract minimal or optimal amounts of physical activity and non-sitting time. For this reason, the committee decided to recommend an amount of physical activity that concurs with other international guidelines and to give only a general guideline to reduce sedentary behaviour [2–4, 56–59].

The guidelines are used for education purposes in the Netherlands. They are similar for children and adolescents and for younger and older adults, because there was insufficient evidence to differentiate. However, when implementing the guidelines in practice, education messages regarding physical activity should be further tailored to for example specific age groups, socioeconomic groups and groups with different activity levels.

### Limitations to our approach

The aim of this study was to derive physical activity guidelines for the general Dutch population. Our search therefore excluded patient-only samples. There is a growing literature, particularly in cancer and diabetes, showing that ‘exercise=medicine’ meaning that physical activity can generate health benefits in a variety of patient groups or reduce the negative effects of disease or its treatment [80, 81] Although we acknowledge that our recommendations cannot be generalized to each and all patient populations they should be considered meaningful for many specific patient groups.

A second limitation is the exclusive use of published meta-analyses and systematic reviews. No attempt was made to go back to the primary literature and redo the meta-analyses or systematic reviews on the original single cohort studies or RCTs. This would have allowed the committee to select high quality and high powered studies only and/or explore specific amounts of physical activity. Such a major undertaking would not have been compatible with the time frame of the Health Council and its requestor, the Minister of Health. As most of the meta-analyses and systematic reviews that the committee has used followed the IOM, PRISMA, or MOOSE guidelines for meta-analyses and systematic reviews, the committee presumes them of sufficient quality.

By far the largest limitation in deriving the guidelines was that the majority of the included studies are based on self-reported physical activity and sedentary behaviour. These enable to rank subjects, but are inappropriate when it comes to determining the actual amounts of physical activity and sedentary time [13, 14, 82, 83]. The committee would therefore argue for public health organisations to adopt regular population-based monitoring of physical activity using accelerometers in addition to questionnaires [84, 85]. This recommendation also holds for research into the health effects of physical activity and sedentary behaviour, which would greatly benefit from objective monitoring. The committee expects that the ongoing wave of new research in which physical activity is measured both objectively (quantity) and subjectively (type and quality) will provide better estimates of the type and actual amount of physical activity required for health. Future updates of the guidelines are, therefore, considered an important mission for public health authorities.

## Conclusions

Prospective studies provide convincing evidence in support of the 2017 Dutch physical activity guidelines and plausible evidence for the sedentary behaviour guidelines. There is insufficient evidence for quantifying the amount of physical activity required for health effects. Research based on a combination of objectively and subjectively-assessed physical activity and sedentary behaviour is needed for more specific guidelines.

## Additional file

Additional file 1: Decision tree for drawing conclusions on the level of evidence for effects (RCTs ) and associations (cohort studies). (PPTX 104 kb)

## Abbreviations

BMI: Body Mass Index
RCTs: Randomized-Controlled Trials
