# Supplementary material for: The 2017 Dutch Physical Activity Guidelines
Source: Int J Behav Nutr Phys Act. 2018 Jun 25;15:58. doi: 10.1186/s12966-018-0661-9 (PMC6016137; doi:10.1186/s12966-018-0661-9)
Supplement: Supplementary file 1 — Decision tree for drawing conclusions on the level of evidence for effects (RCTs ) and associations (cohort studies). (PPTX 104 kb) [file 12966_2018_661_MOESM1_ESM.pptx]

## Slide 1
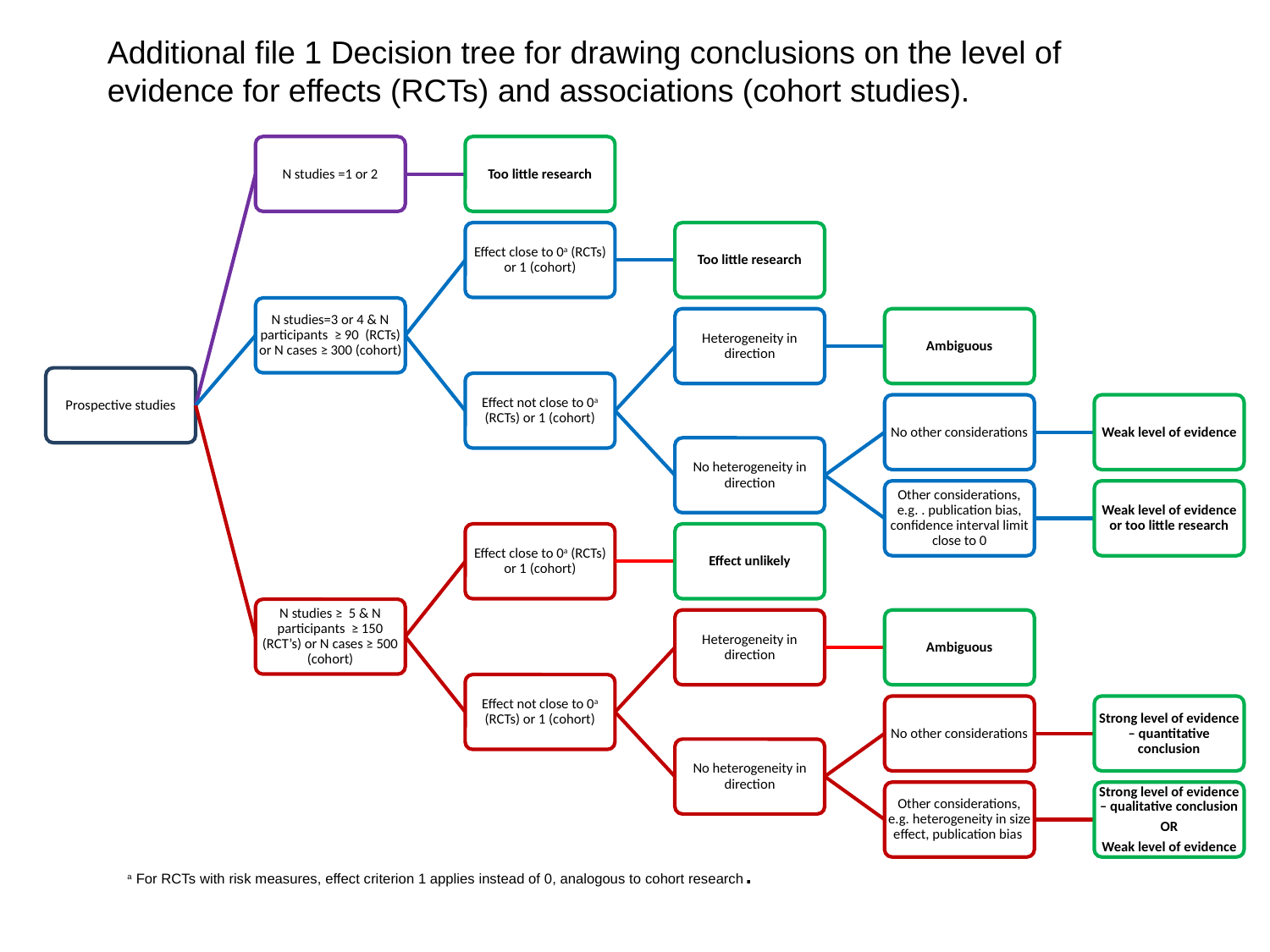

Additional file 1 Decision tree for drawing conclusions on the level of evidence for effects (RCTs) and associations (cohort studies).
a For RCTs with risk measures, effect criterion 1 applies instead of 0, analogous to cohort research.
